# *In Vitro* and *In Vivo* Anti-infective Potential of Thymol Against Early Childhood Caries Causing Dual Species *Candida albicans* and *Streptococcus mutans*

**DOI:** 10.3389/fphar.2021.760768

**Published:** 2021-11-19

**Authors:** Arumugam Priya, Anthonymuthu Selvaraj, Dass Divya, Ramalingam Karthik Raja, Shunmugiah Karutha Pandian

**Affiliations:** ^1^ Department of Biotechnology, Alagappa University, Science Campus, Karaikudi, India; ^2^ Department of Microbiology, Alagappa University, Science Campus, Karaikudi, India

**Keywords:** dual species, thymol, anti-infective, antivirulence, early childhood caries, *C. albicans*, *S. mutans*, *G. mellonella*

## Abstract

Early childhood caries (ECC), a severe form of caries due to cross-kingdom interaction of *Candida albicans* and *Streptococcus mutans*, is a serious childhood dental disease that affects majority of the children with poor background. The present study investigated the anti-infective potential of thymol against *C. albicans* and *S. mutans* dual species for the management of ECC. Thymol, a plant derivative of the monoterpene group, has been well known for its numerous biological activities. Thymol at 300 μg/ml concentration completely arrested growth and proliferation of dual species of *C. albicans* and *S. mutans.* Rapid killing efficacy of pathogens, within a span of 2 min, was observed in the time kill assay. In addition, at sub-inhibitory concentrations, thymol effectively diminished the biofilm formation and virulence of both *C. albicans* and *S. mutans* such as yeast-to-hyphal transition, hyphal-to-yeast transition, filamentation, and acidogenicity and acidurity, respectively, in single and dual species state. qPCR analysis was consistent with virulence assays. Also, through the invertebrate model system *Galleria mellonella*, *in vivo* toxicity and efficacy of the phytocompound was assessed, and it was found that no significant toxic effect was observed. Moreover, thymol was found to be proficient in diminishing the infection under single and dual state in *in vivo* condition. Overall, the results from the present study illustrate the anti-infective potential of thymol against the ECC-causing dual species, *C. albicans* and *S. mutans*, and the applicability of thymol in medicated dentifrice formulation*.*

## Introduction

The oral microbiome of humans comprises more than 700 different species of microorganisms, including bacteria, fungi, mycoplasma, viruses, archaea, and protozoa (Marsh and Zaura, 2017). These communities of microorganisms interact with each other and persist in the oral surfaces as multispecies biofilms. Of the various kinds of interaction between these microbial communities, cross-kingdom interaction between bacteria and fungi is of great interest as it is associated with dental caries (tooth decay) and mucosal infections (Koo et al., 2018). Interaction between *Candida albicans* and *Streptococci* stands as the most common fungal and bacterial communication in the oral cavity. Coinfection with *C. albicans* and oral streptococci species is pronounced with enhanced virulence of dental caries and oropharyngeal diseases (O’Donnell et al., 2015; Allison et al., 2016). More precisely, this interspecies communication ensues in early childhood caries (ECC). ECC has been reported to be the most common childhood oral disease that extremely affects the poor and minority children of age less than 6 years all over the world (Dye et al., 2012; Kassebaum et al., 2015). This severe form of caries is characterized with massive and painful destruction of teeth. Carbohydrate-rich diet such as sucrose elevates the disposition of microbial communities with predominance of aciduric and cariogenic microorganisms. Consequently, enhanced virulence leads to furtherance of dental tissue destruction. *Streptococci* species such as *S. gordonii*, *S. oralis*, and *S. sanguinis* interact with *C. albicans* and subsist with enhanced bacterial colonization and biofilm formation. Typically, in a healthy oral environment, no interaction between *S. mutans* and *C. albicans* is encountered nor no colonization of *C. albicans* is observed in the teeth surface (Xiao et al., 2018). One of the prime factors that contribute to the severe destruction of teeth in ECC is the extended consumption of sucrose-rich foods and beverages, which is due to the increased physical coadhesion between *C. albicans* and *S. mutans* as well as colonization on the tooth surface. The enzyme glycosyltransferases secreted by *S. mutans* bind with the cell surface of *C. albicans* and foster conversion of sucrose to extracellular polysaccharides (EPS), which further provides a binding site for *S. mutans* (Ikono et al., 2019)*.* This unusual interaction further increases the localized microbial burden, acidurity, and production of the extracellular matrix. Eventually, this mixed-kingdom interaction leads to sever tooth decay (Falsetta et al., 2014).

Dual species interaction of *C. albicans* and *S. mutans* is found in the ECC (Marchant et al., 2001; de Carvalho et al., 2006; Raja et al., 2010) and in bracket materials (Rammohan et al., 2012). Also, *S. mutans* and *C. albicans* have been found together in carious lesions (Vílchez et al., 2010). ECC is a severe and aggressive form of caries where *C. albicans* was found in around 96% of caries-positive children and only in 24% of caries-free children (Raja et al., 2010). Dental plaque was found to contain both *S. mutans* and *C. albicans* in about 25.5% healthy individuals (Ribeiro et al., 2012). Also, ECC is a familial disease as this is infectious and transmissible (Douglass and Clark, 2015). As this cross-kingdom interaction increases the virulence of this disease through enhanced biofilm formation, the therapeutic intervention most often fails to completely eradicate the infection. Currently available treatments with synthetic antimicrobials include the use of chemical biocides such as hydrogen peroxide and chlorhexidine, which are demonstrated to be incapable of destroying the infectious organisms beyond the well-formed matrix material (Autio-Gold, 2008; Koo et al., 2017). Moreover, the use of these synthetic antimicrobials ensues in adverse side effects. To circumvent these limitations, the present study demonstrated the use of bioactive molecule derived from natural source as an effective alternate for the treatment of ECC.

In traditional medicine, *Thymus vulgaris* (thyme) has been used for the treatment of various ailments owing to its broad spectrum of pharmacological properties (Amiri, 2012). The major constituent of the thyme essential oil is thymol, which is a phenol monoterpene compound (Burt, 2004; Nickavar et al., 2005; Amiri, 2012). This bioactive molecule is the natural derivative of cymene and structural isomer of carvacrol. It is known to have various biological properties such as antibacterial, antifungal, antioxidant, anticancer, and cognitive-enhancing activities (Tohidpour et al., 2010; Azizi et al., 2012; Braga, 2005). As carvacrol and thymol are generally considered as safe for human consumption, these bioactive molecules are being employed in dental applications (Ogaard et al., 1997; Khan et al., 2017; Kachur and Suntres, 2020). The phenolic hydroxyl group in the chemical structure of thymol is known to confer its biological activities (Nagoor Meeran et al., 2017). Though thymol has been reported to possess antimicrobial activity against various pathogenic organisms including *Staphylococcus aureus*, *Escherichia coli*, *Salmonella Typhimurium*, *C. albicans*, *S. pyogenes*, etc (Braga et al., 2008; Xu et al., 2008; Palaniappan and Holley, 2010), the efficacy of thymol in inhibiting the dual species *C. albicans* and *S. mutans*, the role players in the development of ECC, was unexplored. Thus, the present study investigated the antimicrobial and anti-infective potential of this phytocompound against the growth, biofilm, and other virulence attributes of mono and dual species of *C. albicans* and *S. mutans* for the employability of thymol in the treatment options of ECC.

## Materials and Methods

### Ethical Statement

The saliva sample used in this study was collected from healthy volunteers after obtaining written informed consent. The protocol for experimentation and the use of saliva was assessed and approved by the Institutional Ethical Committee, Alagappa University, Karaikudi (IEC Ref No: IEC/AU/2018/5). Methods followed were carried out in accordance with the appropriate guidelines and regulations.

### Microbial Strains and Growth Conditions

*Streptococcus mutans* UA159 and *Candida albicans* (ATCC 90028) were used in this study. Culturing mono species of *S. mutans* and *C. albicans* (2 × 10^6^ cfu/ml) was performed using THYES (Todd Hewitt broth supplemented with 1% of yeast extract and sucrose) (HiMedia, India) and YPD (1% yeast extract, 2% peptone, and 2% dextrose) broth (HiMedia, India), respectively. For culturing of dual species (equal volume of each culture), TSBS (soybean casein digest medium supplemented with 1% sucrose) medium (HiMedia, India) was used. Cultures were incubated at 37°C for 24 h.

### Phytochemical Stock Solution

Thymol was commercially procured from Alfa Aesar, India. Stock solution of thymol was prepared as 50 mg/ml concentration in methanol (Sigma-Aldrich, India) and stored at room temperature. The highest volume of the compound used was chosen as the volume of methanol to be added for vehicle control in each assay.

### Determination of Minimum Inhibitory Concentration (MIC) and Minimum Microbicidal Concentration (MMC)

The MIC of thymol against *C. albicans* and *S. mutans* was evaluated through microbroth dilution method according to CLSI guidelines (Wikler, 2006; Barbara et al., 2017). For determination of MMC, single and dual species culture of *C. albicans* and *S. mutans* was cultured in the absence and presence of thymol at MIC and sub-MICs. After 24 h of incubation at 37°C, the control and treated groups were subjected to serial dilution followed by spotting and spread-plating on appropriate agar plates (Priya et al., 2021).

### Time Kill Assay

Time kill assay was performed to analyze the short-term microbicidal effect of thymol on single and dual species culture of *C. albicans* and *S. mutans* as described by Niu et al. (2020) with slight modifications*.* Briefly, 2 × 10^6^ cells were taken for mono species, and an equal volume of *C. albicans* and *S. mutans* was taken for dual species. Various concentrations of thymol (1X, 2X, 5X, and 10X MIC) were added separately. After 2-min exposure, the compound activity was restrained by removal through two rounds of centrifugation. Cells were resuspended in phosphate buffered saline (PBS) and serially diluted, and 5 µl each from all the serial dilutions was also spotted on agar plates.

### Effect on Biofilm Formation

*C. albicans*, *S. mutans*, and dual species cultures in the absence and presence of thymol at MIC and sub-MICs were allowed to form biofilm on 1 cm × 1 cm glass surface for 24 h at 37°C. Post incubation, the glass slides were carefully removed from the medium, dip-washed in sterile PBS to remove loosely bound cells, air-dried, and stained with 0.4% crystal violet. Biofilm cells in the stained glass sections were visualized under a light microscope (Nikon Eclipse 80i, United States) at a magnification of ×400 and documented.

### Effect of Thymol on Biofilm Adherence

In addition to microscopic observation of the single and dual species *C. albicans* and *S. mutans* biofilm under the influence and absence of thymol, cell viability assay was performed with resazurin dye (Alamar blue). Alamar blue is a versatile metabolic dye, which is a redox indicator that is reduced within the cell due to cellular metabolism. Single and dual species cultures of *C. albicans* and *S. mutans* were allowed to form biofilm in the presence and absence of thymol at MIC and sub-MICs (32.5, 75, 150, and 300 μg/ml) on polystyrene surface. At the end of 24 h, the planktonic cells were discarded, and loosely bound cells were removed by careful washing with PBS. Surface-attached cells were then resuspended in PBS solution. Stock solution of Alamar blue (Sigma-Aldrich, India) at a concentration of 6.5 mg/ml was prepared in 1× PBS. To 0.9 ml of cell suspension in PBS, 0.1 ml of Alamar blue was added and incubated in the dark for 4 h at 37°C. Sterile PBS added with Alamar blue substrate alone was maintained as blank. Samples were centrifuged at 8,000 rpm for 10 min after incubation. Supernatant was collected, and the fluorescent intensity was measured at 590-nm emission and 560-nm excitation wavelengths (Muthamil et al., 2020).

### Effect of Thymol on Biofilm Formation in the Presence of Saliva

Unstimulated whole saliva (UWS) was collected from healthy individuals with good oral hygiene. Prior to the collection of saliva, the volunteers were refrained from eating, drinking, and brushing for 2 h. The saliva sample was collected by the method of spitting into a sterile tube, which was immediately clarified by centrifugation at 4,000 × *g* for 10 min. The cell debris were removed, and the supernatants were pooled and stored at −20°C until use. For biofilm formation, 200 µl of cell suspensions (single and dual species) was added with 20 µl of clarified saliva in the absence and presence of thymol. After 24 h of incubation, the planktonic cells were discarded, and loosely bound cells were washed off with sterile PBS. Surface-bound biofilm cells were stained with 0.4% crystal violet and subsequently destained with 15% glacial acetic acid solution, the absorbance of which was read at 570 nm using a multifunctional spectrophotometer (Spectra Max 3, Molecular Devices, United States) (Ahn et al., 2008).

### Effect of Thymol on Hyphal Morphogenesis of *C. albicans*

The impact of thymol on fungal morphogenesis between yeast and hyphal forms was analyzed through the following assays (Priya and Pandian, 2020).

#### Yeast-to-Hyphal Transition

*C. albicans* and dual species of *C. albicans* and *S. mutans* were cultured in a YPD medium supplemented with 10% FBS in the absence and presence of thymol at MIC and sub-MICs at 37°C for 4 h under constant shaking at 160 rpm. Following incubation, morphological transitions in the cells were observed under a microscope (Nikon Eclipse Ts2R, Japan).

#### Hyphal-to-Yeast Transition

*C. albicans* in single and dual species state was allowed to form hyphae by incubating in an RPMI medium for 4 h at 37°C with constant shaking at 160 rpm. Subsequently, thymol at various concentrations was added, further incubated for 2 h, and visualized under a microscope.

#### Filamentous Morphology

Spider agar (1% of mannitol, 0.2% of dipotassium hydrogen phosphate, 1% of nutrient broth, and 1.8% agar) supplemented with 5% FBS was added with MIC and sub-MICs of thymol. An agar plate with an appropriate volume of methanol (0.6%) served as the control. After solidification, 5 µl of *C. albicans* culture in single and dual species state was spotted on the center of agar plates and incubated at 37°C for 5 days.

### Effect of Thymol on Acidogenicity and Acidurance of *S. mutans*

#### Glycolytic pH Drop Assay

*S. mutans* cells cultured under single and dual state were harvested by centrifugation at mid-logarithmic phase and washed in PBS. The cell pellets were then resuspended in a salt solution comprising 50 mM potassium chloride and 1 mM magnesium chloride in the absence and presence of various concentrations of thymol, and the pH of the mixture was adjusted to 7.2 with 0.2 M potassium hydroxide. To this, glucose at 1% w/v final concentration was added, and decline in the pH level was monitored for a period of 120 min at 15-min intervals.

#### Acid Tolerance Assay

The effect of thymol on acid tolerance mechanisms of *S. mutans* in the single and dual species state was appraised with the viable count of cells following exposure to two different acidic pH conditions. Cells cultured in the absence and presence of thymol at sub-MICs were pelleted by centrifugation. Cell pellets from the control and each treatment group were split into two aliquots, unadapted and adapted cells, of which the former was directly resuspended in the THYES broth of pH 3.5, incubated at 37°C for 2 h, and the latter was initially suspended in THYES broth of pH 5.5 for 1 h followed by exposure to lethal pH 3.5 for 2 h. Subsequently, viable cells from adapted and unadapted groups were enumerated by spread plating. Dilutions were also spotted on agar plates (Priya et al., 2021).

### Post Antimicrobial Effect

*C. albicans* and *S. mutans* cells in single and mixed state were subjected to a brief exposure of thymol (1X, 2X, 5X, and 10X MIC) for 1 h after which the compound was removed by centrifugation. Appropriate positive controls were maintained in parallel. For mono species of *C. albicans* and *S. mutans*, amphotericin B (MIC: 2.5 μg/ml) and chlorhexidine (MIC: 16 μg/ml) were used, respectively. For dual species, both amphotericin B and chlorhexidine were used in combination. Post exposure, 1% culture from each group was used as the inoculum and cultured in an appropriate medium. Changes in the cell density were spectrophotometrically observed for a period of 12 h with 1-h time interval (Taweechaisupapong et al., 2012).

### Ability of *C. albicans* and *S. mutans* to Develop Resistance Against Thymol

#### Spontaneous Resistance Assay

The cell density of the overnight cultures of *C. albicans*, *S. mutans* and dual species was adjusted to 1 × 10^8^ cells. Cultures were spread-plated on agar plates with various concentrations of thymol and incubated at 37°C for 48 h. Cultures plated on agar plates devoid of thymol served as the control (Min et al., 2017).

#### Successive Passage Assay

Initially, the cultures were exposed to the lowest concentration of thymol, and at subsequent days, the cells were passaged and exposed to increasing concentrations until MIC. After every passage, the cell density was measured spectrometrically by reading absorbance at 600 nm (Hua et al., 2010).

### Effect of Thymol on Expression of Key Virulence Genes

Total RNA from *C. albicans*, *S. mutans*, and dual species culture was extracted by the Trizol method. Using a high-capacity cDNA Reverse Transcription Kit (Applied Biosystems, United States), the extracted RNA was converted to cDNA. qPCR analysis was performed with the SYBR Green Master Mix (Applied Biosystems, United States) for candidate genes (list of genes, primer details, and function are provided in Table 1) of *C. albicans* and *S. mutans.* Changes in the expression were calculated by the ^Δ Δ^CT method (Livak and Schmittgen, 2001).

**TABLE 1:** List of candidate genes, their role in virulence, and pathogenicity and primer details.

S. No	Gene	Function	Primer sequence (5′–3′)	
			Forward	Reverse
1	*eap1*	Cell adhesion, filamentation, and invasion. Mediates adhesion to polystyrene and epithelial cells	TGT​GAT​GGC​GGT​TCT​TGT​TC	GGT​AGT​GAC​GGT​GAT​GAT​AGT​GAC​A
2	*hwp1*	Hyphal development, biofilm formation. Promotes yeast adhesion to epithelial cells	GCT​CCT​GCT​CCT​GAA​ATG​AC	CTG​GAG​CAA​TTG​GTG​AGG​TT
3	*ras1*	Cell adhesion, filamentous growth, induction, and maintenance of hyphae, white-opaque switching	CCC​AAC​TAT​TGA​GGA​TTC​TTA​TCG​TAA​A	TCT​CAT​GGC​CAG​ATA​TTC​TTC​TTG
4	*als1*	Cell surface adhesin. Important for adhesion to oral mucosa. Mediates yeast aggregation	CCT​ATC​TGA​CTA​AGA​CTG​CAC​C	ACA​GTT​GGA​TTT​GGC​AGT​GGA
5	*ece1*	Hyphal specific protein, Candidalysin. Mediates adhesion, biofilm formation, and filamentation	CCA​GAA​ATT​GTT​GCT​CGT​GTT​GCC​A	TCC​AGG​ACG​CCA​TCA​AAA​ACG​TTA​G
6	*nrg1*	Transcriptional repressor of filamentous growth. Repress *ece1* and *hwp1*	CCA​AGT​ACC​TCC​ACC​AGC​AT	GGG​AGT​TGG​CCA​GTA​AAT​CA
7	*ume6*	Transcriptional regulator of filamentous growth. Important for hyphal elongation and germ tube formation	ACC​ACC​ACT​ACC​ACC​ACC​AC	TAT​CCC​CAT​TTC​CAA​GTC​CA
8	*tup1*	Transcriptional repressor, farnesol-mediated inhibition of filamentation, regulates phenotypic switching	CTT​GGA​GTT​GGC​CCA​TAG​AA	TGG​TGC​CAC​AAT​CTG​TTG​TT
9	*efg1*	Transcriptional regulator for switch between white and opaque cells. Required for biofilm formation, filamentation. Regulator of cell wall dynamics	GCC​TCG​AGC​ACT​TCC​ACT​GT	TTT​TTT​CAT​CTT​CCC​ACA​TGG​TAG​T
10	*hst7*	Required for opaque mating or white biofilm formation	TCA​TCA​GCT​TCT​TCT​ATA​C	TAT​TGA​GGA​AAT​GAC​AGT​T
11	*cph1*	Transcription factor involved in pseudohyphal and hypha formation and phenotypic switching	TAT​GAC​GCT​TCT​GGG​TTT​CC	ATC​CCA​TGG​CAA​TTT​GTT​GT
12	*vicR*	Two-component regulatory system. Regulates cell wall biogenesis and biofilm formation	TGA​CAC​GAT​TAC​AGC​CTT​TGA​TG	CGT​CTA​GTT​CTG​GTA​ACA​TTA​AGT​CCA​ATA
13	*gtfB*	Glucosyltransferases synthesizing water-insoluble glucan from sucrose	AAA​GCA​ACG​GAT​ACA​GGG​GA	CTC​TGT​CAT​TGG​TGT​AGC​GC
14	*gtfC*	Glucosyltransferases synthesizing water-soluble and -insoluble glucans	GGT​TTA​ACG​TCA​AAA​TTA​GCT​GTA​TTA​GC	CTC​AAC​CAA​CCG​CCA​CTG​TT
15	*gtfD*	Glucosyltransferases synthesizing water-soluble glucan synthesis	GAA​GTA​TGG​CGG​TGC​TTT​CC	ATA​ACC​AAC​ACC​ACG​GCC​TA
16	*gbpB*	Glucan binding protein. Contributes to sucrose-dependent biofilm formation	ATG​GCG​GTT​ATG​GAC​ACG​TT	TTT​GGC​CAC​CTT​GAA​CAC​CT
17	*smu0630*	Hypothetical protein involved in biofilm formation, cell separation, and autolysis	GTT​AGT​TCT​GGT​TTT​GAC​CGC​AAT	CCC​TCA​ACA​ACA​ACA​TCA​AAG​GT
18	*comDE*	Competence stimulating peptide. Regulation of bacteriocin production and competence	ACA​ATT​CCT​TGA​GTT​CCA​TCC​AAG	TGGTCTGCTGCCTGTTGC

### Evaluation of *In Vivo* Efficacy of Thymol

The toxic effect of thymol, if any, and the *in vivo* efficacy to clear the *C. albicans* and *S. mutans* infection were analyzed through the invertebrate animal model *Galleria mellonella.* Larvae weighing around 0.2–0.4 g were taken for experiments. Ten larvae were taken per group. A total of 2 × 10^6^ and 2 × 10^4^ cells of *C. albicans* and *S. mutans*, respectively, were taken for infection. Thymol at 300 mg/kg was injected for both toxicity analysis and treatment groups. Injection was performed with a U-100 insulin syringe (Dispovan, HMD, India) in the last proleg. For survival analysis, nine different groups were segregated. Group I received PBS alone and served as the injection control. Group II received PBS along with 2% methanol and served as the vehicle control. Group III larvae were injected with thymol (300 mg/kg) for analysis of the toxicity. Groups IV–VI were designated as the infection control and received the cultures *C. albicans*, *S. mutans*, and dual species, respectively. An appropriate volume of culture was taken in the U-100 syringe and injected on the last left proleg. Groups VII–IX were designated as the treatment group where the larvae received thymol in addition to infection. Thymol was injected on the last right proleg. Larva groups were incubated at 37°C for 5 days. Survival was monitored every 12 h. For *in vivo* efficacy of thymol in controlling the infection, three larvae from infected and treated groups were cut open with a scalpel; the content was suspended in sterile PBS, and the serial dilutions were plated on a selective medium (HiChrome *candida* differential medium (HiMedia, India) for *C. albicans*; Mitis salivarius agar (HiMedia, India) for *S. mutans*; for dual species, both the plates were used) (Selvaraj et al., 2020).

### Statistical Analysis

All the experiments were carried out in at least three biological replicates with at least two technical replicates, and values are presented as mean ± standard deviation (SD). To analyze the significant difference between the value of control and treated samples, one-way analysis of variance (ANOVA) and Duncan’s *post hoc* test were performed with a significant *p*-value of <0.05 by the SPSS statistical software package version 17.0 (Chicago, IL, United States).

## Results

### MIC and MMC of Thymol Against Single and Dual Species of *C. albicans* and *S. mutans*

**FIGURE 1:**
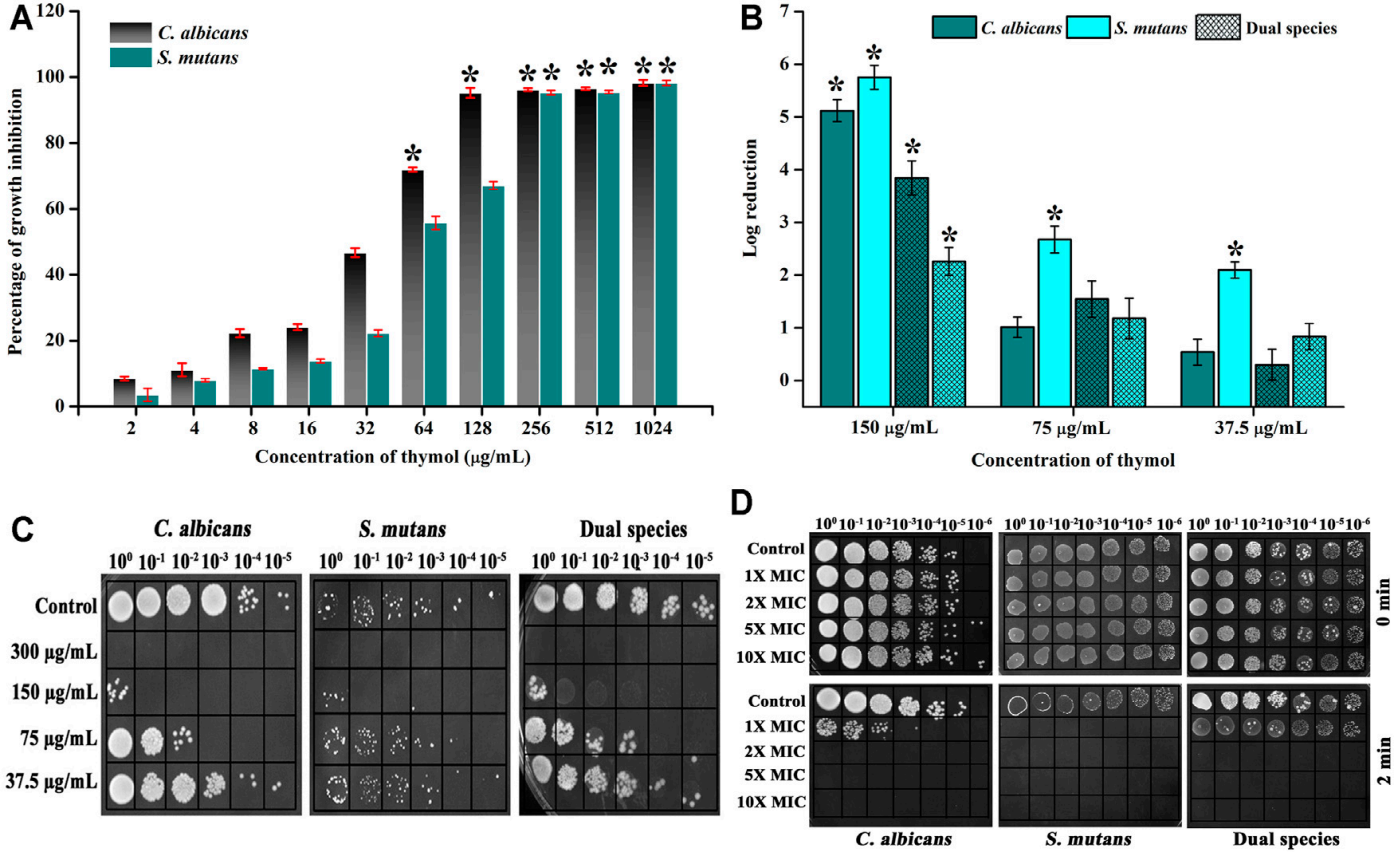
Impact of thymol on the growth of mono and dual species of *C. albicans* and *S. mutans.* **(A)** Thymol significantly impaired the growth of *C. albicans* and *S. mutans* at 128 and 256 μg/ml concentrations, respectively. **(B)** Proliferation of growth was completely arrested at 300 μg/ml for single and dual species of *C. albicans* and *S. mutans.* **(B)** Log reduction in cfu/ml of pathogens under sub-inhibitory concentrations of thymol. **(C)** Spot assay confirming the complete inhibition of growth at 300 μg/ml and reduction in microbial load with increasing concentrations. **(D)** Rapid killing efficiency of thymol. Two minutes exposure of thymol completely inhibited *S. mutans* at 1X MIC and *C. albicans* and dual species at 2X MIC. Error bars represent standard deviations from the mean and * indicates significance *p* < 0.05.

Initially, the MIC of thymol was assessed against single species of *C. albicans* and *S. mutans* through microbroth dilution assay. It was found that for monoculture, thymol at 128 and 256 μg/ml inhibited the visible growth of *C. albicans* and *S. mutans*, respectively (Figure 1A). Hence, for dual species, 300 μg/ml of thymol was analyzed for growth inhibitory effect, and the same concentration was found to be effective in inhibiting the growth of dual species. Thus, 300 μg/ml of thymol was considered to be the MIC and MMC for dual species. Through cfu analysis, it was evident that thymol exerts the growth inhibitory effect against single and dual species of *C. albicans* and *S. mutans* in a concentration-dependent manner (Figure 1B). Spot assay displays that thymol at 300 μg/ml completely inhibited the growth of single and dual species of *C. albicans* and *S. mutans*, and a concentration-dependent growth inhibition can also be witnessed (Figure 1C).

### Effect of Brief Exposure of Thymol on Viability of Single and Dual Species *C. albicans* and *S. mutans*

As the end application of this study is directly related to the dentifrice formulation, the impact of limited time exposure of bioactives on these pathogens was analyzed through time kill assay where the microbes were exposed to thymol for 2 min. At MIC, *S. mutans* cells were completely killed by the action of thymol, whereas for *C. albicans* and dual species, 2X MIC cleared the viable cells (Figure 1D).

### Biofilm Inhibitory Effect of Thymol at Sub-Inhibitory Concentrations

**FIGURE 2:**
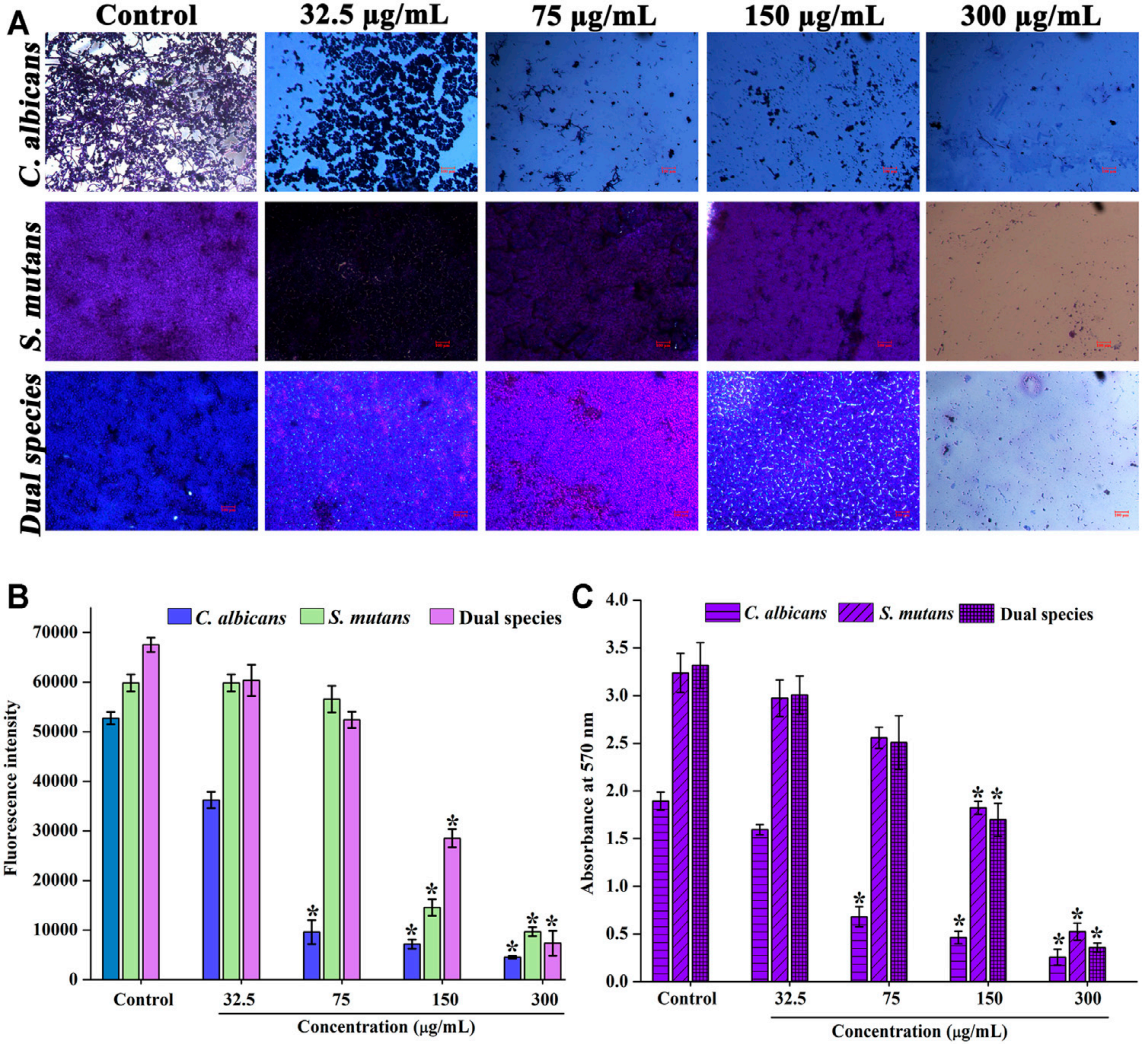
Effect of thymol on the biofilm of single and dual species of *C. albicans* and *S. mutans.* Concentration-dependent biofilm inhibitory effect of thymol **(A)** as visualized through a microscope **(B)** Alamar blue assay **(C)** biofilm inhibitory effect of thymol in the presence of saliva. Error bars represent standard deviations from the mean and * indicates significance *p* < 0.05.

The impact of sub-inhibitory concentrations of thymol was microscopically appraised. Dose-dependent diminution in the surface adherence of cells was observed for thymol treatment. Under single and dual species state, the biofilm formation and surface adherence of *C. albicans* were impaired in a concentration-dependent manner by the influence of thymol. In addition to reduction in surface adherence, the hyphal form was also found to be arrested. At MIC, the viability of *S. mutans* was completely lost, and thus, no surface adherence of *S. mutans* was found (Figure 2A).

Similarly, metabolic viability assay was performed for the biofilm of *C. albicans* and *S. mutans* single and dual species under the influence of thymol. Results observed are in line with the microscopic observation, as *C. albicans* biofilm adherence was found to be diminished in a concentration-dependent manner under both single and dual species condition (Figure 2B).

The proficiency of thymol in inhibiting the biofilm formation of *C. albicans* and *S. mutans* in the presence of saliva was also analyzed. The efficiency of thymol continued to be the same even in the presence of saliva (Figure 2C). These results suggest that thymol can be effective against the *C. albicans* and *S. mutans* biofilm.

### Reduction in the Virulence Attributes of *C. albicans* in Single and Dual Species State Under the Influence of Thymol

**FIGURE 3:**
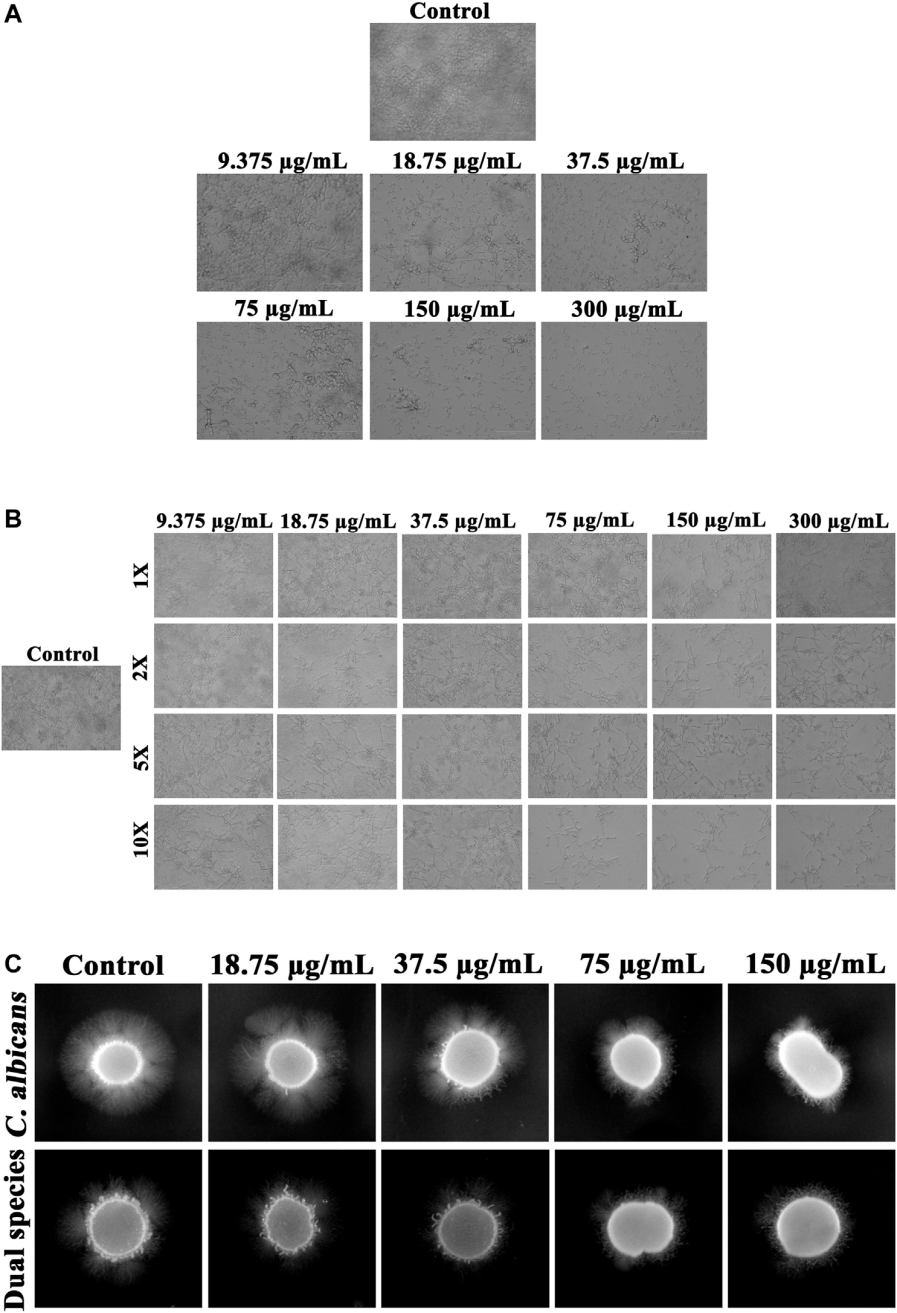
Influence of thymol on fungal morphogenesis of *C. albicans* in single and dual species state. **(A)** Thymol, in a concentration-dependent manner, suppressed the transition of yeast cells to hyphal form. **(B)** Transition of hyphal cells to yeast morphogenesis was augmented by increasing concentrations of thymol. **(C)** Filamentation with the supplement of FBS was efficiently repressed by thymol in both single and dual species state.

Phenotypic switch between yeast and hyphal forms under the influence of thymol was analyzed. In a concentration-dependent manner, thymol could restrain the shift of yeast to hyphal phase (Figure 3A) and can revert the hyphal cells to yeast morphogenesis (Figure 3B). Hyphal morphogenesis of *C. albicans* during interaction with *S. mutans* was found to be diminished, and the same has been evidenced in the present study through the filamentation assay, which when compared to mono species, the filamentation of *C. albicans* under dual state was found to be less. Despite the single or dual state, thymol significantly impeded the development of filamentous morphology (Figure 3C).

### Decline in the Acidogenic and Aciduric Potential of Single and Dual Species *S. mutans* Under the Effect of Thymol

**FIGURE 4:**
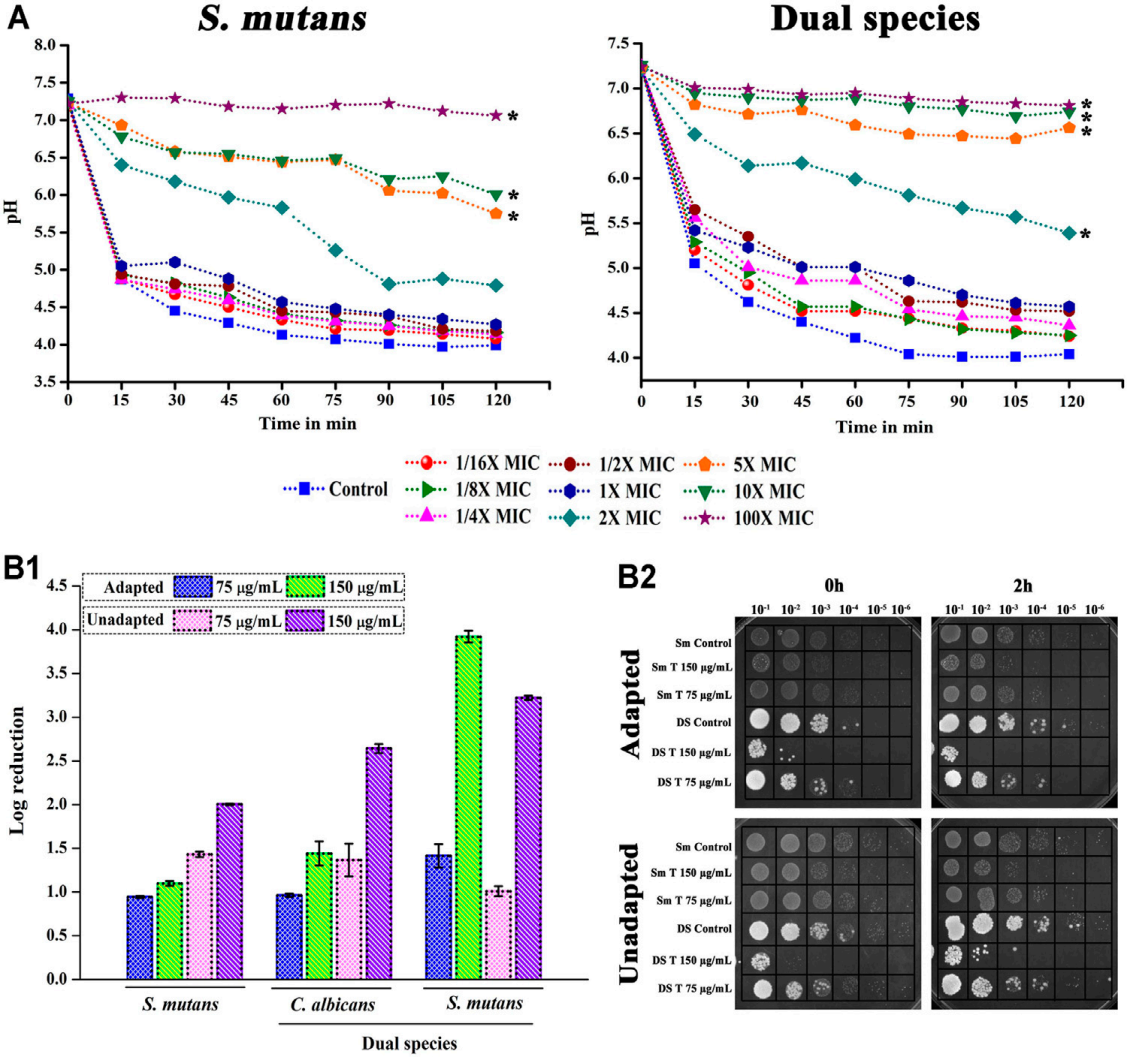
Effect of thymol on acidogenicity and acidurance of *S. mutans* in single and dual species state. **(A)** Due to the interference of thymol in the glycolytic pathway, decline in the metabolism of glucose resulting in decreased acid production was observed in both single and dual species culture. **(B)** Thymol deteriorates the acid tolerance mechanism of *S. mutans.* **(B-1)** Log reduction in cfu/ml of pathogens habituated in acidic condition under the influence of thymol; **(b-2)** spot assay confirming the reduction in microbial load. Error bars represent standard deviations from the mean and * indicates significance *p* < 0.05.

Metabolic breakdown of carbohydrate through glycolysis was interfered by the presence of thymol. In single as well as dual state, the pH of the control was dropped to more acidic condition. For thymol treatment, at MIC, a slight variation in the pH change was noted, whereas at higher MICs a significant change was observed (Figure 4A).

Correspondingly, the aciduric ability of *C. albicans* and *S. mutans* was found to be significantly diminished under the impact of thymol. Both adapted and unadapted cells were found to be sensitized to the acidic pH condition under the single and dual species state when treated with thymol (Figure 4B). Unadapted cells were found to be more sensitive to thymol. Irrespective of prior adaptation conditions, thymol reduced the survival of cells under low pH condition, which is an added advantage for the treatment of caries.

### Post Antimicrobial Effect of Thymol

**FIGURE 5:**
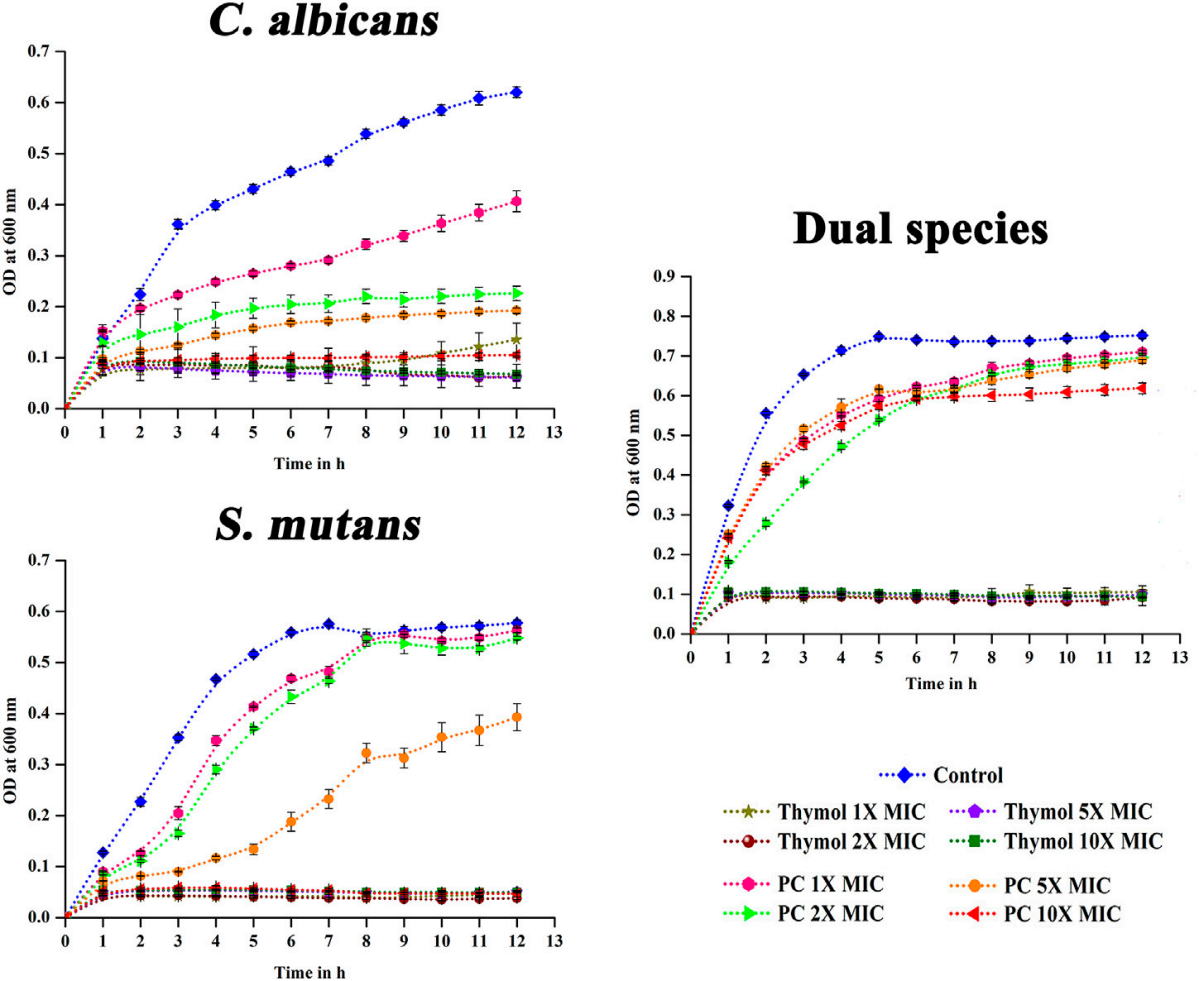
Post antimicrobial effect of thymol. Brief exposure to 1X–10X MIC of thymol significantly suppressed the proliferation of the pathogens in single as well as dual state, whereas positive controls did not exhibit significant post antimicrobial effect. PC, positive control. Amphotericin B (MIC- 2.5 μg/ml) and chlorhexidine (MIC- 16 μg/ml) were used as the positive control for *C. albicans* and *S. mutans*, respectively. For dual species, amphotericin B and chlorhexidine were used in combination. Error bars represent standard deviations from the mean.

As oral pathogens are exposed to dentifrices only for a short duration, the antimicrobial effect after the removal of thymol was analyzed. Compared to the positive controls—chlorhexidine and amphotericin b—thymol exhibited proficient post antimicrobial effect against single and dual species of *C. albicans* and *S. mutans* even at MIC by arresting the proliferation of cells (Figure 5).

### Diminished Possibility for Resistance Development by *C. albicans* and *S. mutans* Against Thymol

**FIGURE 6:**
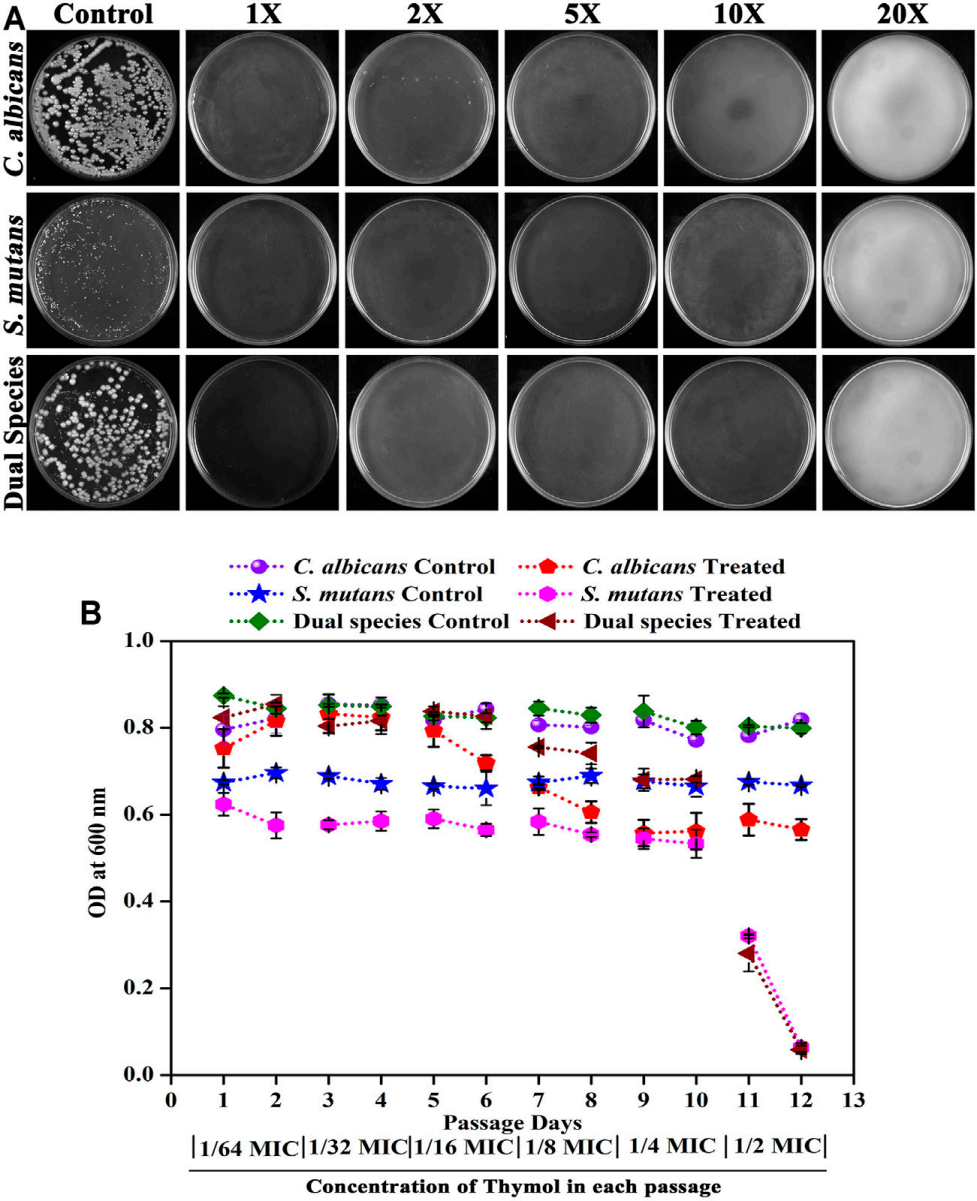
Assessment of the ability of the pathogens to develop resistance against thymol. **(A)** Spontaneous resistance development. Both single and dual species of *C. albicans* and *S. mutans* did not develop spontaneous resistance to thymol even at 20X MIC. **(B)** Resistance development in successive passage. Subculturing of pathogens with increasing concentrations of thymol did not initiate resistance development. Error bars represent standard deviations from the mean.

The possibility of resistance development against thymol by single and dual species of *C. albicans* and *S. mutans* was investigated. Spontaneous resistance (Figure 6A) to higher concentration of thymol as well as resistance to successive passage (Figure 6B) in the presence of increasing concentrations of thymol were not acquired by the pathogens. When single and dual species of *C. albicans* and *S. mutans* was allowed to grow on a medium supplemented with high concentrations of thymol, no colonies were developed, signifying that the pathogens were unable to outgrow in the presence of thymol. Similarly, when the pathogens were exposed to thymol from lower concentration to higher concentration over a period, no resistance development was observed as complete growth inhibition was observed at sub-MIC of thymol at the 12th day of passage (Figure 6B).

### Dynamics in the Expression of Candidate Virulence Genes

**FIGURE 7:**
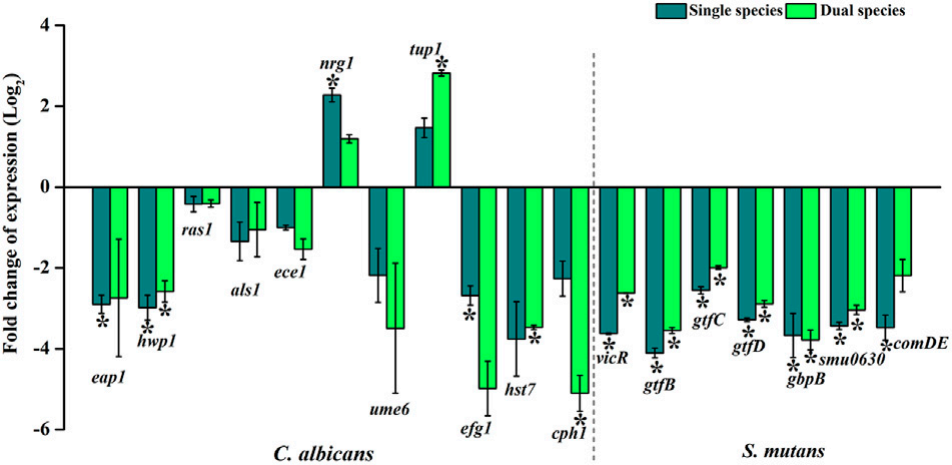
Gene expression profiling of candidate genes associated with virulence and pathogenesis of *C. albicans* and *S. mutans* under single and dual species state. Under the influence of thymol, *C. albicans* genes that are responsible for biofilm formation, hyphal and filamentous development, adhesins, and morphological phenotypic switching are found to be downregulated. Negative transcriptional regulators of filamentation such as *nrg1* and *tup1* were found to be upregulated. *S. mutans* genes that are allied with biofilm formation, competence, glucan synthesis, which mediates interaction between *C. albicans* and *S. mutans*, is downregulated by the impact of thymol. Downregulation of virulence genes validates the anti-infective efficacy of thymol. Error bars represent standard deviations from the mean and * indicates significance *p* < 0.05.

Treatment with thymol influenced the transcriptional level modulations in the virulence genes (Figure 7). Except for the transcriptional repressors *nrg1* and *tup1*, the expression of all other genes of both *C. albicans* and *S. mutans* was found to be downregulated. Expressions of *nrg1* and *tup1* were found to be upregulated, which is in line with the antihyphal activity observed through *in vitro* assays. Genes involved in the development and maintenance of hyphae in *C. albicans* such as *hwp1*, *ras1*, *ece1*, and *cph1*, genes responsible for filamentous morphology such as *eap 1*, *efg1*, adhesin *als 1*, and the transcriptional regulator of filamentous growth such as *ume6* and *hst7*, which is required for biofilm formation, are found to be downregulated. Negative transcriptional regulators of filamentation such as *nrg1* and *tup1* were upregulated upon thymol treatment in both single and dual species. Downregulation of genes associated with the hyphal development and filamentous morphology and upregulation of negative regulators of the same under the influence of sub-inhibitory concentration of thymol imply that the compound can influence crucial virulence aspects of the pathogen. Similarly, *vicR* and *comDE*, the two major two-component regulatory systems of *S. mutans*, were found to be downregulated. Downregulation of *vicR* has affected the expression of glucosyltransferases *gtfBCD*, which are responsible for the synthesis of water-soluble and -insoluble glucans that are elemental bridge molecules between bacteria and acquired pellicle, thereby facilitating the colonization of the microbial biofilm. Decreased expression of *ComDE*, the two-component signal transduction system allied with the quorum sensing, which is known to regulate the competence and biofilm formation of *S. mutans*, suggests that the communication between the microbial systems resulting in the increased biofilm amalgamation has been impaired by the action of thymol. Similarly, the expression of two other genes *gbpB* and *Smu0630* that are correlated with biofilm formation are declined.

### *In Vivo* Rescuing Potential of Thymol From *C. albicans* and *S. mutans* Infection

**FIGURE 8:**
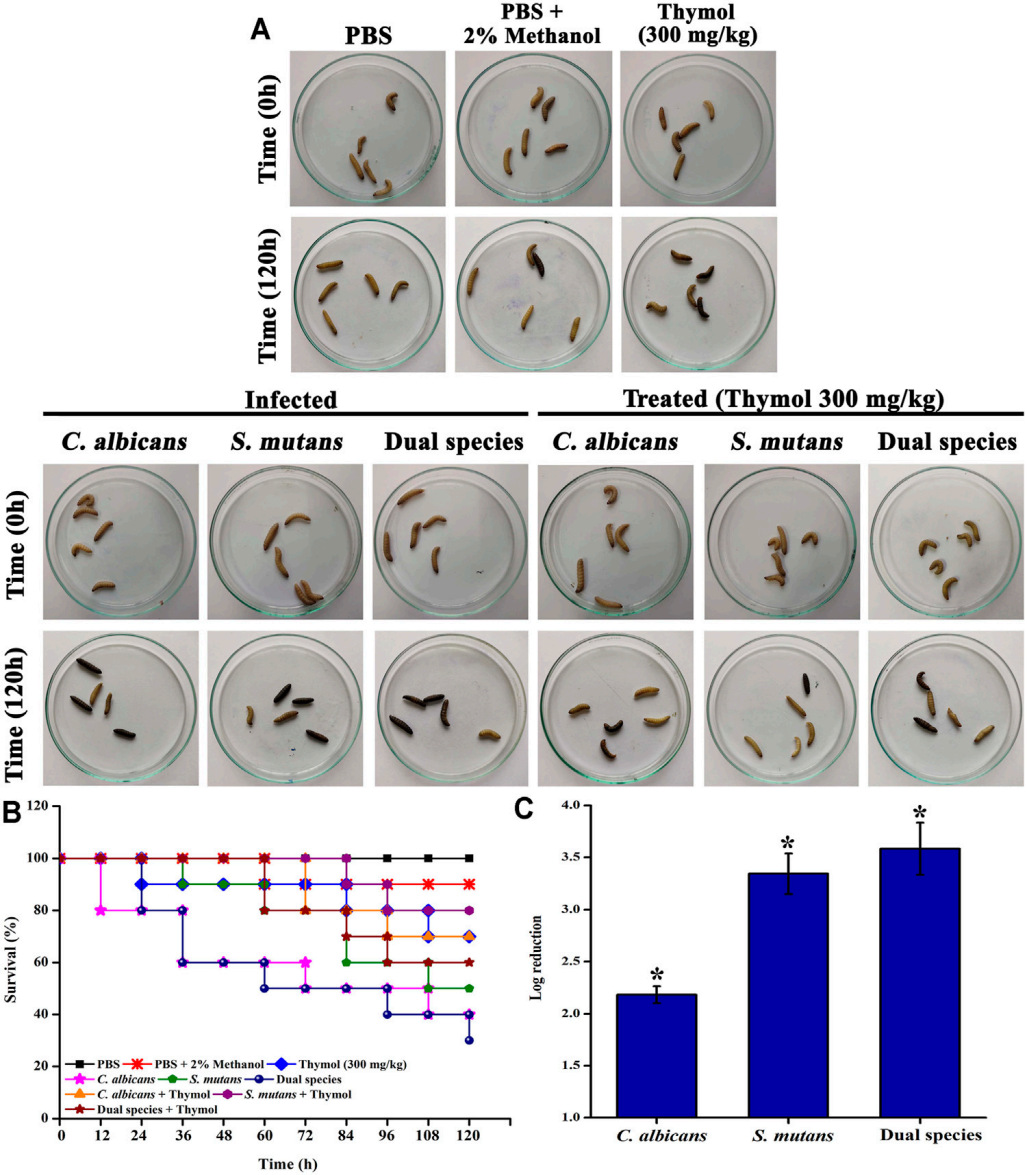
*In vivo* anti-infective efficacy of thymol against single and dual species of *C. albicans* and *S. mutans* in the *G. mellonella* model system. **(A)** Representative image displaying the survival status of larvae at time 0 h and time 120 h in different groups. Thymol at 300 μg/ml concentration was administered to check the toxic effect. No significant reduction in mortality was observed. Hence, thymol at the tested concentration was found to be nontoxic. Infected groups received respective cultures, and the treated groups received both culture and thymol at 300 μg/ml concentration. Larvae that turned to complete black and no response to physical stimulus were considered to be dead. **(B)** The Kaplan−Meier survival plot displaying the survival of *G. mellonella* under the influence of various treatments. Thymol at 300 mg/kg was found to be nontoxic to the *G. mellonella* larvae. *C. albicans* and dual species infection drastically reduced the survival rate, whereas thymol treatment rescued *G. mellonella* from infection. **(C)** Reduction in the internal microbial burden due to the anti-infective potential of thymol. Error bars represent standard deviations from the mean and * indicates significance *p* < 0.05.

Thymol at 300 mg/kg concentration does not exert any significant toxic effect to the larvae, whereas infection with *C. albicans*, *S. mutans*, and dual species of *C. albicans* and *S. mutans* impaired the survival (Figures 8A,B). Treatment with thymol rescued the larvae from the infection and increased the survival rate (Figure 8C). About 70% of larvae survived up to 120 h after administration of thymol. Only 35, 50, and 30% of larvae survived following infection with *C. albicans*, *S. mutans*, and dual species, respectively. On the other hand, treatment with thymol increased the survival rate to 70, 80, and 60% in larvae infected with *C. albicans*, *S. mutans*, and dual species, respectively. In addition to this, the *in vivo* infection clearance was also promoted by thymol treatment, which was evidenced through the reduced colony count in CFU analysis.

## Discussion

Amid the numerous infectious diseases, dental caries is represented as one of the most prevalent chronic diseases that affect majority of the people all over the world (Dye et al., 2007; Selwitz et al., 2007). Individuals who encounter this infection once are susceptible to infectivity throughout their lifetime (Featherstone, 2000; Pitts, 2004). Dental caries rises from the impaired balance between the availability of minerals in the teeth and colonization of oral microbial community as biofilms (Fejerskov, 2004; Scheie and Petersen, 2004). Thus, interaction between the acid-producing bacteria and fermentable carbohydrates remains as a principal underlying machinery in the progression of teeth erosion (Philip et al., 2018). ECC, a virulent form of dental caries that affects the primary tooth, is also affiliated with the increased consumption of fermentable carbohydrates accompanied with improper bottle-feeding practices (de Carvalho et al., 2006; Phantumvanit et al., 2018). Various other risk factors that are associated with ECC are environmental risk factors, dietary risk factors, and microbiological risk factors. A later predisposing factor is the principal etiological cause for the development and progression of ECC (Kawashita et al., 2011). Co-occurrence of *C. albicans* and *S. mutans* is frequently detected from the plaque sample of ECC (de Carvalho et al., 2006). Restoration or surgical removal of the carious teeth is the established therapeutic intervention in the current setting. Nevertheless, the relapse of the caries around the restored teeth or extent to the nearby teeth is very frequently reported (Berkowitz, 2003; Graves et al., 2004). Numerous reports are available on the epidemiology, etiology, prevention measures, and association between *C. albicans* and *S. mutans* in the disease progression (de Carvalho et al., 2006; Falsetta et al., 2014; Koo and Bowen, 2014; Lobo et al., 2019). Not too many reports are available regarding therapeutic interventions to confine this cross-kingdom alliance (Bombarda et al., 2019; Li et al., 2019). In order to decline this obscurity, the present study investigated the therapeutic efficacy of thymol against the major virulence attributes of *C. albicans* and *S. mutans* during their solitary and cohabitation. Thymol is a major phytocompound of the thyme species that has been used for various pharmacological purposes for decades. Biological activities of thymol are not limited to antioxidant, anti-inflammatory, antibacterial, antifungal, antiseptic, and antitumor activities (Nagoor Meeran et al., 2017). Here, in the present study, the anti-infective efficacy of thymol against the dual species of *C. albicans* and *S. mutans* was analyzed. Initial experiments with the determination of MIC and MMC signified that thymol at 300 μg/ml concentration completely inhibited the growth and proliferation of single and dual species of *C. albicans* and *S. mutans.* In addition to growth inhibition effect, the proficiency to kill the existing mass of cells within a span of 2 min implies the therapeutic efficiency of thymol.

Synergistic interaction between *C. albicans* and *S. mutans* within the carious biofilm ensues in enhanced virulence of both the pathogens. Also, several studies report that the presence of *C. albicans* supports the extensive colonization of *S. mutans* in the dental biofilm*.* Thus, the impact of sub-inhibitory concentrations of thymol on biofilm formation of single and dual species of *C. albicans* and *S. mutans* was microscopically appraised, and a dose-dependent diminution in the surface adherence of cells was observed.

Furthermore, the impact of thymol on major virulence attributes of *C. albicans* and *S. mutans* was reviewed. Previous *in vitro* and *in vivo* studies have shown that the hyphae of *C. albicans* can penetrate the enamel, dentinal tubules, and root canal in the large caries lesions (Şen et al., 1997; Jacob et al., 1998; Waltimo et al., 2000). Fungal morphogenesis and filamentation conditions were found to be controlled by thymol. Similarly, *S. mutans* has been shown to produce acid from the dietary carbohydrates (acidogenicity) and able to survive under lethal pH condition (acidurity), which is one of the most imperative attributes for the progression of dental caries. *C. albicans* can also produce acids and survive under acidic pH. Accordingly, the influence of thymol on glycolytic pH drop and acid tolerance was measured for *S. mutans* under the single and dual species state. Both the acidogenic and aciduric ability of *S. mutans* was found to be impaired by thymol.

Along with the ability to restrain the major virulence attributes of *C. albicans* and *S. mutans*, thymol also displayed post antimicrobial efficacy, which was found to be superior to the positive controls.

When a pathogen is frequently exposed to a growth-suppressing agent, the development of resistance may arise as a consequence of natural selection. However, pathogens did not develop resistance against thymol, which could be due to the fact that this bioactive regulated various genes/transcriptional regulators of both the organisms. This further strengthens the application of thymol in treating ECC.

Additionally, the decreased expression of genes that are directly associated with virulence and pathogenesis of *C. albicans* and *S. mutans* under both single and dual species state by thymol alludes the anti-infective efficacy against these ECC-causing pathogens.

*Galleria mellonella* is an invertebrate model organism that has been used to study pathogenicity, host–pathogen interaction, immune response to microbial infections, and toxicity. Allegra et al. (2018) reported that *G. mellonella* can discriminate between toxic and nontoxic chemicals, and this model system is a better tool than the cell culture system. There are several other studies that have shown the nontoxic nature and *in vivo* efficacy of their compound in *G. mellonella* (Lu et al., 2019; Merigo et al., 2017; Desbois and Coote, 2011; Gibreel and Upton 2013 etc). Rossoni et al. (2019) reported *G. mellonella* as an experimental model system to study oral pathogens and detailed about the studies that used the *G. mellonella* model system to study oral pathogens, which include *C. albicans* and *S. mutans*. There are also several studies that demonstrated the usefulness of the *G. mellonella* model system to study the virulence of *C. albicans* (Mesa-Arango et al., 2013; Kavanagh and Sheehan, 2018; Bergin et al., 2006 etc). Numerous studies have employed *G. mellonella* to study the virulence of *S. mutans* (Alves et al., 2020; Abranches et al., 2008; Avilés-Reyes et al., 2014; Miller et al., 2015). Reports are also available on studies related to dual species in the *G. mellonella* model system (Kean et al., 2017; Sheehan et al., 2020 etc). Based on this background, *G. mellonella* was expended as a model system in this study to evaluate the toxicity and *in vivo* efficacy of thymol. Thymol at 300 mg/kg concentration does not exert any significant toxic effect to the larvae, whereas infection with *C. albicans*, *S. mutans*, and dual species of *C. albicans* and *S. mutans* impaired the survival. Treatment with thymol rescued the larvae from the infection and increased the survival rate.

As the primary aim of this investigation is to evaluate the proficiency of thymol against the ECC-causing dual species *C. albicans* and *S. mutans*, the practical applicability of thymol in prophylaxis/treatment is crucial. Dental caries, which is the buildup of microbial plaque on the teeth surface, can be controlled by certain mechanical self-care oral hygiene practices such as tooth brushing and dental flossing. Improper oral hygiene practices and recalcitrant nature of the microbial biofilm result in recurrent and persistent infection. A broad range of oral care products in different forms such as toothpastes, mouthwashes, medicated chewing gum, etc. is available in the market. In addition to the basic purpose of the dentifrices, certain products are specifically used for the control of infectious organisms. More precisely, antiplaque mouthwashes are being commercialized excessively. These products were produced to contain synthetic or natural actives with antimicrobial activity (Jacobsen et al., 2001). Rather than the use of synthetic and chemical agents with side effects, bioactive components from the natural sources can be a better alternative. In the recent decade, research on the formulation and development of herbal-based toothpastes and mouthwashes has been accelerated. Currently, chewing gum has been progressing toward an effective drug delivery system rather than a candy. In addition to application in drug delivery for systemic infections, chewing of sugar-free gums can have added benefits to oral health such as their cleaning ability, reduction of conditions such as dry mouth, increasing the pH of the biofilm, and remineralization of enamel (Wessel et al., 2016). As ECC is primarily associated with children, proper usage of toothpaste or mouth rinse cannot be guaranteed. Thus, the authors consider that medicated chewing gum formulation with thymol will be the best for the prevention/treatment of ECC.

## Data Availability

The original contributions presented in the study are included in the article/Supplementary Material. Further inquiries can be directed to the corresponding author.
